# Phytochemical-Induced Metabolic Modulation: Dihydroartemisinin Regulates Cellular Metabolism in Madin-Darby Bovine Kidney Cells

**DOI:** 10.3390/ijms26104531

**Published:** 2025-05-09

**Authors:** Jindong Gao, Kuang Lei, Mengdi Zhang, Jinhua Yin, Changmin Hu

**Affiliations:** 1 College of Animal Science and Technology, Tarim University, Alar 843300, China; jindong-gao@taru.edu.cn (J.G.); 120210031@stumail.taru.edu.cn (M.Z.); 2 Key Laboratory of Tarim Animal Husbandry Science and Technology, Xinjiang Production and Construction Corps, Alar 843300, China; 3 College of Veterinary Medicine, Huazhong Agricultural University, Wuhan 430070, China; agaki@webmail.hzau.edu.cn

**Keywords:** dihydroartemisinin (DHA), phytopharmacology, herbal bioactive compounds, cellular metabolic remodeling, metabolomic profiling

## Abstract

Dihydroartemisinin (DHA) is a bioactive phytopharmaceutical with diverse pharmacological potential, predominantly because of its established antiplasmodial efficacy. Here, we investigated the effects of DHA on metabolic homeostasis in Madin-Darby bovine kidney cells in the context of dose-specific adaptation of metabolism and regulation of biochemistry pathway changes. According to our findings, extensive changes in metabolism were revealed by PCA, accounting for a variability of 59.4% to distinguish contrasting metabolic signatures from normal cells. Metabolomic characterization demonstrated 67 constituting metabolites of baseline cellular processes, while 32 and 44 metabolites have demonstrated differential abundance in low- and high-dose treatments, respectively. Impaired metabolism of glycerophospholipid, amino acid, and nucleotide biosynthesis was reported with implications such as regulation of membrane reorganization, nitrogen metabolism, and cellular bioenergetics. Bioindicators of high-volume lysophosphatidylcholine (18:0) and choline phosphate revealed a lipid homeostatic change, in addition to imbalances in glutamic acid and proline levels. Pathway regulation further modulated ABC transporters and arachidonic acid signaling as implications of systemic phytopharmaceutical-modulated reorganization of metabolism. Hence, the study gives mechanistic insight into DHA-initiated modulation of cellular metabolism in MDBK cells, elucidating its status as a cellular metabolism regulator. Findings confirm the potential of DHA as a phytopharmaceutical in modulating diseases of metabolism, further solidifying its relevance in evidence-based traditional herbal remedies and natural compound therapeutics.

## 1. Introduction

With the rising demand for animal products worldwide, it has become increasingly important to unravel the complex interactions underlying livestock health and productivity. Metabolism is a basic physiological process of life needed to meet energy requirements for maintenance, growth, and reproduction. This extensive system comprises numerous vital organs that synchronize energy production and resource utilization to uphold physiological equilibrium [1,2]. The kidney is one such vital organ participating in the maintenance of internal homeostasis. Its cellular machinery is in a state of dynamic flux in response to a wide variety of stimuli, securing the excretion of waste products, the maintenance of fluid balance, and biochemical synthesis [3,4]. Such metabolic changes may be an entry point toward the elucidation of the molecular mechanisms that underlie resilience and adaptability in livestock, at least in an experimental setup emulating naturally occurring stresses. Metabolic disturbances, caused by stress, constitute one of the key constraints to the sustainability of livestock. Changes in nutrition, climate, and other environmental stresses can all reshape the cellular pathways that define animal health with a loss of efficiency and heightened vulnerability. Understanding how cells, particularly renal cells, reprogram metabolism upon such stressors is an important element in addressing these challenges. Advanced metabolomics approaches now make it possible to comprehensively outline these changes and uncover hidden metabolic signatures and pathways inaccessible to traditional studies. This investigation presented here uses the kidney as a focus in addressing such adaptation processes, taking advantage of new analytical techniques that reveal pathways defining metabolic robustness and open up avenues for precision livestock management.

Among the various factors influencing animal health, metabolic health is paramount, as it acts as a core factor for productivity, disease resistance, and welfare. Bovine renal cells are a central model for studying metabolic responses in livestock, as these cells play important roles in many physiological functions, including filtration and regulation of metabolism [5]. Metabolism relies on an intricate network of biochemical pathways that convert food into energy and structural components vital for growth, reproduction, and homeostasis. Numerous external and internal factors, such as variations in diet, environmental conditions, or genetic predispositions, modulate this ever-changing system. Such metabolic pathways, if understood, will lead to better feeding practices, health interventions, and, ultimately, better productivity among livestock. More specifically, the kidneys play a major role in the regulation of metabolism by processing the body’s waste products, maintaining fluid and electrolyte balance, and synthesizing hormones essential for various metabolic processes. Therefore, studying bovine renal cells’ metabolic reactions under different experimental conditions is important in designing strategies to improve the health and productivity of livestock [6].

The importance of understanding metabolic pathways in livestock goes beyond just productivity concerns. Most stressors, such as nutritional deficiencies, disease pressures, and environmental changes, frequently menace the health of livestock [7]. These stressors may cause metabolic dysregulation, which diminishes productivity, impairs immune function, and makes diseases more likely. By elucidating the metabolic pathways affected by these stressors, researchers can identify potential biomarkers that enable early diagnosis and develop targeted nutritional and management strategies to improve metabolic health [8,9]. In recent years, there has been an increased interest in applying metabolomics to the domain of animal science. Therefore, it has been revealed through studies that metabolomics may provide potential knowledge on physiological responses to different stimuli: dietary changes, health challenges, or environmental fluctuations in living organisms [10,11]. Indeed, metabolomics has turned into a powerful tool for a wide range of applications, including the identification of biomarkers for metabolic diseases in livestock, assessment of feed additives aimed at improving animal health and productivity, and study of how environmental stressors affect metabolic pathways. With such advances, most of the literature still emphasizes a few key organs or tissues and does not discuss a comprehensive metabolic characterization of bovine kidney cells. This gap in the literature underlines the need for a wider approach to the understanding of the broader metabolic dynamics of maintaining homeostasis.

The present study was hence undertaken with a view to fill this gap and contribute meaningfully to animal science and livestock management regarding critical metabolites implicated in key metabolic pathways, i.e., lipids, amino acids, and nucleotides. These metabolic pathways are related to overall metabolic health; therefore, responses of these pathways against various experimental treatments may highlight the adaptive mechanisms of bovine kidney cells and their implications in livestock management. The expected outcomes of this study contribute to a deep understanding of metabolic adaptation in the kidney cell and provide useful insight. This is particularly timely and relevant in light of contemporary challenges to the livestock industry, in terms of high production costs, increased scrutiny with respect to animal welfare regulation, and the appeal towards sustainable production practices. Therefore, this investigation represents a basic description of metabolic responses in bovine kidney cells under an experimental setup, made possible by detecting biomarker metabolites and their respective pathways. By means of advanced metabolomics techniques, we try to reveal the complex biochemical network beneath metabolic health in livestock. This will help fill in the gaps that have been identified in the knowledge on bovine kidney metabolism and hopefully lay a foundation for future studies on improving the health and welfare of livestock. The novelty of this study resides in its systemic metabolomic investigation of DHA-induced metabolic disturbances in MDBK cells—a cell culture model not previously exposed to such phytochemical interrogation. Unlike previous research with a systemic or non-renal tissue focus, our investigation is the first to examine dose-dependent biochemical alterations in lipid, amino acid, and nucleotide metabolism in renal epithelial cells using high-resolution LC-MS-based metabolomics.

## 2. Results

### 2.1. Metabolomic Analysis Reveals Significant Alterations in Metabolic Profiles Following Dihydroartemisinin (DHA) Treatment

**Figure 1:**
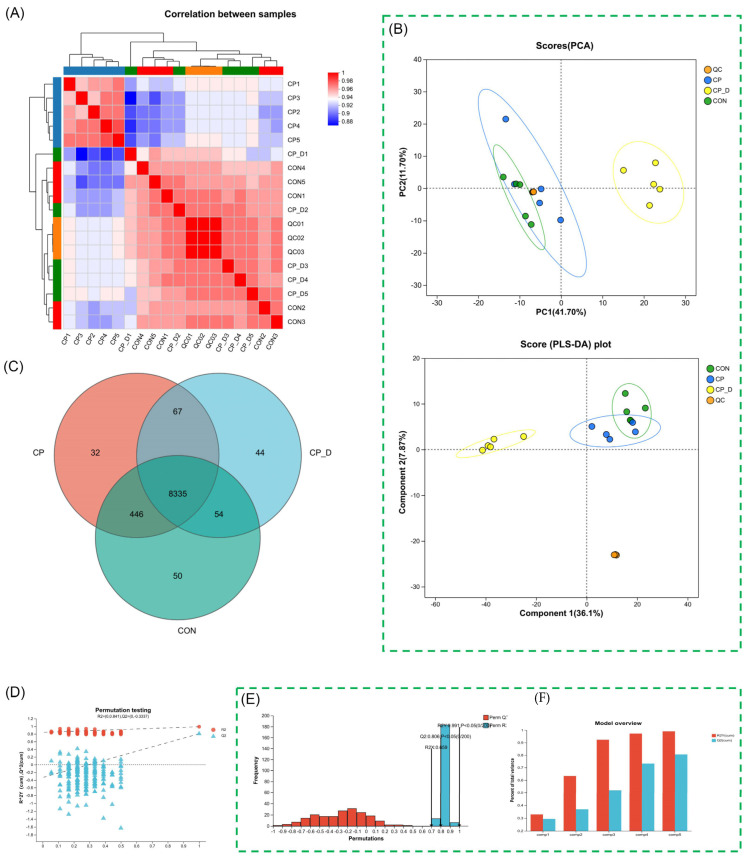
Univariate and multivariate comparison of metabolic profiles following DHA treatment. (**A**) Heatmap of sample correlations with hierarchical clustering showing different metabolic profiles by treatment groups. (**B**) PCA plot of variance between groups, where principal components 1 (41.7%) and 2 (17.7%) separate treated (CP, CP_D) from control (CON) samples. (**C**) PLS-DA plot of dose-dependent metabolite changes, with minimal overlap between groups. (**D**) Venn diagram depicting common and unique metabolites of CP, CP_D, and CON with 67 common features pointing towards baseline metabolic pathways. (**E**) Permutation test for statistical robustness of PLS-DA model where R^2^ (0.841) and Q^2^ (0.3337) values significantly exceed permuted data. (**F**) Bar chart of explanatory (R^2^Y) and predictive (Q^2^) power of PLS-DA indicating clear dose-dependent metabolic pattern differences.

The heatmap showed similarities and differences between groups, indicating the metabolic profiles of CP and CP_D groups are more similar, thus reflecting a treatment-dependent response. It showed negative correlations between the treated and untreated groups, hence suggesting that the intervention has dramatically changed the metabolomics profile. Also, hierarchical clustering and subclusters within the treatment groups suggested an intragroup variability due to biological noise or subtle subpopulations of metabolites. The heatmap represented the consistency and reproducibility of QC samples acting as controls of the analytical pipeline (Figure 1A). Principal component analysis (PCA) of metabolite data revealed 41.7% and 17.7% of the variance within principal component 1 and principal component 2, respectively. There was a clear clustering of CP, CP_D, CON, and QC samples, reflecting the distinct metabolic pattern coupled with the experimental condition. QC sample clusters were arranged tightly together near the origin, reinforcing experimental and analytical consistency. CON samples (controls) occupied a distinct region, separated from CP and CP_D, indicating that the metabolomic profiles of untreated samples were fundamentally different from treated ones. CP and CP_D groups were spatially distinct, suggesting that the dose-dependent treatment induced progressive metabolic shifts. The CP_D group exhibited a greater spread along PC1 and PC2, likely reflecting a broader metabolic response compared to CP. PC1 likely represents treatment-induced metabolic alterations, while PC2 may correspond to other sources of variance, such as inter-individual variability. This separation highlights that treatment drives the primary variance in the dataset, suggesting a hypothesis-driven mechanistic influence on metabolic pathways.

PLS-DA plots offer enhanced resolution of the metabolic differences between experimental groups. CP, CP_D, CON, and QC samples showed distinct clustering with minimal overlap, indicating that the metabolomic features are well separated by treatment condition. The CP_D group demonstrates the most pronounced divergence, highlighting a stronger or broader metabolic response under dose-dependent treatment. The separation along component 1 (36.1%) and component 2 (27.8%) supports the hypothesis of a significant treatment effect. The ability of PLS-DA to separate CP and CP_D groups suggested that specific metabolites are likely upregulated or downregulated in a dose-dependent manner, making them potential biomarkers for treatment efficacy. The tight clustering of QC samples confirms the technical precision of the experiment, while the distinct CON cluster emphasizes the metabolic shift induced by treatment (Figure 1B,C). The Venn diagram provides a quantitative comparison of shared and unique metabolites across CP, CP_D, and CON groups. CP (32 unique metabolites), CP_D (44 unique), and CON (50 unique) groups show treatment-specific metabolomic fingerprints. These unique features are indicative of condition-specific metabolic processes triggered by the intervention. The 67 metabolites shared among all three groups likely represent baseline metabolic pathways essential for maintaining cellular homeostasis, regardless of treatment conditions. The unique metabolites in CP_D compared to CP suggest dose-dependent activation or inhibition of specific metabolic pathways. The greater overlap between CP and CP_D (446 shared features) indicated that both groups share a core response to treatment, albeit with gradations in intensity (Figure 1D).

A histogram of permuted R^2^ and Q^2^ values was compared to the actual model. The observed R^2^ (0.841) and Q^2^ (0.3337) values of the model were far superior to the permuted data, ensuring the statistical validity of the PLS-DA model (Figure 1E). These metrics define the capability of the model to explain (R^2^) and predict (Q^2^) the variance in the dataset. Strong R^2^ and Q^2^ values indicated that the metabolic changes induced by treatment were true factors and not artifacts of random noise. A confidence factor for using the PLS-DA model in identifying key discriminative metabolites could thereby be obtained. The histogram indicated that none of the permuted Q^2^ values exceed the observed value, thus lending further credibility to the model. Another bar chart summarizes the explanatory power assessed by R^2^Y and predictive power assessed by Q^2^ of the PLS-DA model across experimental groups. High values of R^2^Y in both CP and CP_D groups confirmed that the model holds a substantial portion of the variance induced by the treatment. These values support a significant metabolic shift due to dose-dependent interventions. However, Q^2^ values demonstrated the ability to predict metabolic changes accurately, particularly in the CP_D group, suggesting that the dose-dependent treatment induces a consistent and predictable metabolic response (Figure 1F).

### 2.2. Comprehensive Metabolic Reprogramming Highlights Lipid Dysregulation, Amino Acid Alterations, and Pathway Adaptations

**Figure 2:**
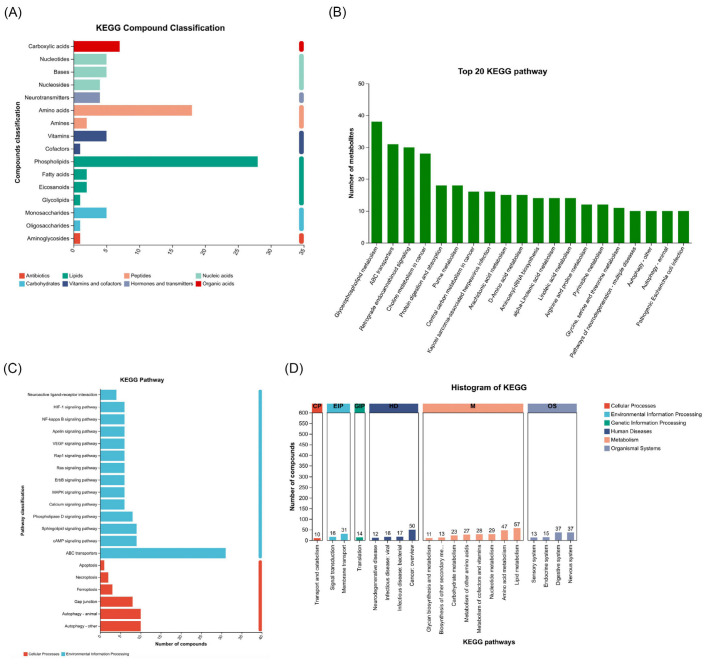
Profiling of comprehensive metabolomic alterations under DHA treatments. (**A**) Compound classification shows that phospholipids, amino acids, and neurotransmitters are the most abundant metabolites. (**B**) KEGG pathway enrichment identified the top 20 pathways with significant enrichment, among which glycerophospholipid metabolism is the most affected. (**C**) Detailed pathway classification showed enrichment related to environmental informational processing, cellular processes, and diseases. (**D**) KEGG pathway histogram reveals the functional distribution of the impacted pathways between cellular processes, environmental information processing, and human disease.

The metabolic landscape under investigation reveals substantial biochemical alterations across multiple compound classes and pathways, providing insights into the systemic reprogramming induced by the treatment. A detailed analysis highlights that phospholipids are the most significantly altered class, reflecting disruptions in lipid metabolism, which are often indicative of membrane remodeling and energy homeostasis perturbations. In addition to phospholipids, notable changes were observed in amino acids, neurotransmitters, and monosaccharides, suggesting a strong impact on protein synthesis, signaling regulation, and energy-related processes (Figure 2A). These results again pinpoint that various classes of metabolites are involved, and the most profoundly disturbed pathways relate to lipids. KEGG pathway enrichment analysis identified the top 20 pathways targeted by the treatment (Figure 2B), in which glycerophospholipid metabolism was the most significantly affected. This pathway is central to cell membrane integrity and signaling, and its dysregulation could reflect adaptive responses to cellular stress or metabolic demands. Additionally, pathways such as ABC transporters and choline metabolism are prominently enriched, suggesting disturbances in metabolite transport and lipid turnover processes. Other pathways, including purine metabolism, amino acid biosynthesis, and arachidonic acid metabolism, further highlight the broad metabolic shifts, encompassing nucleotide turnover, protein metabolism, and inflammatory signaling responses. Notably, pathways such as retrograde endocannabinoid signaling and Kaposin sarcoma-associated herpesvirus infection point toward metabolic adaptations potentially linked to cellular stress, signaling dysregulation, and pathogenic responses.

The classification of impacted pathways reveals a significant dominance of pathways associated with environmental information processing and cellular processes. Pathways like phospholipid signaling, ABC transporters, and MAPK signaling play crucial roles in cellular communication, stress adaptation, and metabolic regulation. Additionally, the enrichment of pathways related to apoptosis and autophagy indicates cellular attempts to manage stress-induced damage and maintain homeostasis. This analysis reveals that the treatment not only alters primary metabolic pathways but also influences higher-order cellular functions, particularly those involved in signaling, transport, and survival mechanisms (Figure 2C).

Histograms can categorize pathways across distinct functional domains: cellular processes, environmental information processing, and human diseases. Among these, metabolite transport, signal transduction, and cancer-related pathways emerge as key functional domains affected by the treatment. For instance, pathways such as cGMP-PKG signaling and VEGF signaling are critical regulators of vascular and cellular homeostasis, further linking the observed metabolic changes to broader physiological adaptations. Notably, the involvement of pathways related to neurotransmitter signaling, such as neuroactive ligand–receptor interactions, highlights the interconnectedness between metabolism and signaling cascades, further emphasizing the systemic impact of the treatment (Figure 2D). Taken together, all these pathways give a clear outline of the metabolic and cellular reprogramming. Pathways associated with lipids, amino acid metabolism, and various signaling cascades are highly enriched, indicating a multifaceted response to treatment that goes from membrane remodeling and adaptation to stress, to energy metabolism. The engagement of apoptotic and autophagic pathways, along with those related to disease, underscores the cellular effort to counteract metabolic derangements in the quest for homeostasis. Taken together, these results provide a global view of the biochemical response to treatment and, hence, shed light on the cross-talk between metabolic and cellular processes in adapting to extrinsic perturbations.

### 2.3. Metabolomic Profiling Reveals Perturbations in Carboxylic Acids, Glycerophospholipids, and Key Differential Metabolic Pathways

**Figure 3:**
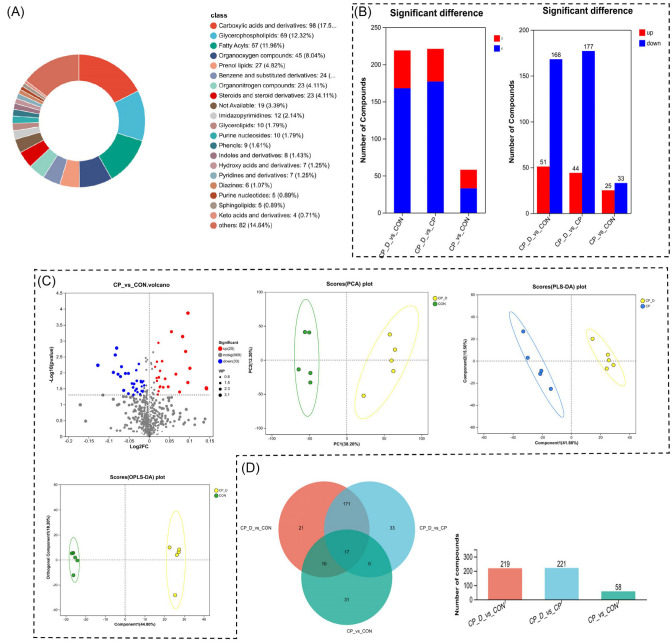
Comparative and quantitative metabolic profiling between treatment groups. (**A**) Metabolite classification indicates glycerophospholipids and carboxylic acids as most prominent, demarcating the role of such metabolites in metabolic reprogramming. (**B**) Bar plots of number of significantly regulated (up- and downregulated) metabolites between pairwise comparisons, CP_0.3_vs_CON, CP_0.9_vs_CON, and CP_0.9_vs_CP, reflecting significant downregulation. (**C**) Volcano plots, PCA, and PLS-DA analyses reflect differences between treatment and control groups, while the volcano plots reflect the predominant up- and downregulating metabolites. (**D**) Venn diagram and bar plot of unique and common metabolites between comparisons as evidence of treatment-unique metabolite profiles.

Metabolomic analysis revealed substantial changes across compound classes, pathways, and specific metabolites, indicating comprehensive metabolic perturbations. In the classification of compounds (Figure 3A), carboxylic acids and derivatives emerged as the most abundant class, followed closely by glycerophospholipids and fatty acids. These three categories accounted for a significant proportion of the detected compounds, suggesting their central role in metabolic regulation under treatment conditions. Other notable compounds included organooxygen compounds and steroids, though their contributions were relatively modest. The presence of diverse compound classes reflects the complexity of metabolic reprogramming and the involvement of multiple biochemical pathways.

To further analyze differential abundance between groups, the quantitative changes in metabolite levels were visualized (Figure 3B). Compounds exhibiting significant differences were classified into upregulated and downregulated groups. Comparisons between control and treatment groups revealed distinct patterns, where the majority of metabolites displayed downregulation, particularly in CP_0.3_vs_CP and CP_0.9_vs_CP comparisons. In contrast, CP_0.9_vs_CON comparisons highlighted more balanced shifts between upregulated and downregulated metabolites. These findings indicate that metabolic suppression is prevalent in certain conditions, while others show selective activation of specific metabolic pathways. To assess clustering and group separation, multivariate analyses, including principal component analysis (PCA) and partial least squares discriminant analysis (PLS-DA), were performed (Figure 3C). PCA scores demonstrated clear separation between the groups, suggesting distinct metabolic profiles. PLS-DA further refined this distinction by improving separation between treatment and control samples, highlighting the robustness of metabolic alterations. The volcano plot identified significantly altered metabolites, with distinct clusters of upregulated (red) and downregulated (blue) metabolites. This clustering reinforces the idea that metabolic shifts occur in response to treatment, with specific metabolites driving the observed changes.

Venn diagram analysis provided insights into shared and unique metabolites across comparisons. A significant number of metabolites were found exclusively in individual comparisons, such as 171 unique compounds in CP_0.3_vs_CON and 33 unique compounds in CP_0.9_vs_CP. However, 17 compounds were consistently shared across all comparisons, emphasizing their pivotal role in the metabolic response (Figure 3D). The quantitative bar chart further highlighted the magnitude of these differences, with a large number of altered compounds in each comparison, reinforcing the widespread metabolic disruption caused by treatment. These results collectively demonstrate that carboxylic acids, glycerophospholipids, and fatty acids are central to the metabolic response, exhibiting dynamic changes in abundance. The observed patterns of upregulation, downregulation, and clustering suggest a coordinated reprogramming of metabolic pathways, likely driven by cellular adaptations to treatment conditions.

**Figure 4:**
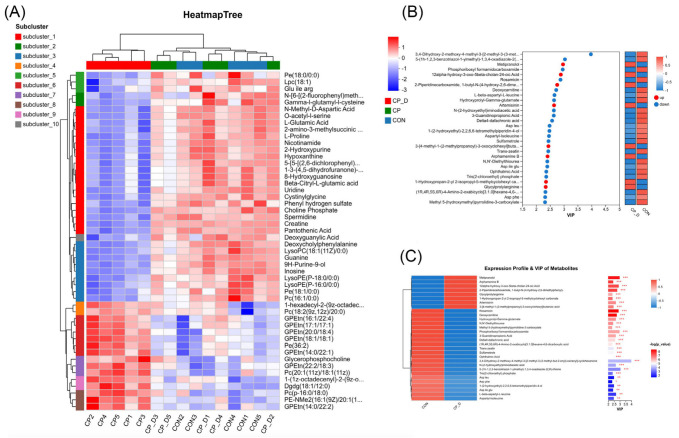
The metabolic profile analysis, highlighting key metabolite variations, group-specific clustering, and significant contributors to the differentiation of experimental groups. (**A**) A hierarchical clustering heatmap representing metabolite expression profile partitions in different subclusters. Different groups, CP_D, CP, and CON, are represented by color intensity, referring to the relative abundance of metabolites. (**B**) VIP (variable importance in projection) scores of PLS-DA analysis showing metabolites that were significant contributors to the differentiation between groups. Upregulated metabolites are colored red, and downregulated metabolites are colored blue. (**C**) Integration of expression profiles with VIP scores for important metabolites. Heatmap of relative expression across groups is integrated with a bar plot of VIP values and significance levels (* *p* < 0.05, ** *p* < 0.01, *** *p* < 0.001).

Metabolic reprogramming plays a pivotal role in understanding the biochemical landscape influenced by therapeutic interventions, and the results revealed compelling evidence of the distinct biochemical signatures associated with varying treatment conditions. The comprehensive metabolic alteration analysis showed distinct biochemical findings for the treatment groups CP_0.3, CP_0.9, and CON. In each subcluster, the hypothesis suggests metabolic reprogramming is a consequence of therapeutic intervention, representing the complex interplay between the administered compounds and the underlying metabolic networks. There were specific metabolites that upregulated in the CP-treated groups compared to controls. These include but are not limited to L-glutamic acid, L-proline, and choline phosphate, each exhibiting important functionality in cellular metabolism. Two of these, L-glutamic acid and choline phosphate, were definitively recognized by direct contrast with reference standards and confirmatory MS/MS spectra. Likewise, lysophosphatidylcholine (18:0) was verified by diagnostic fragment ions and co-elution with a reference standard, thereby validating the integrity of our metabolic interpretations. More importantly, there was a highly significant increase in the level of L-glutamic acid. This amino acid participates in neurotransmitter synthesis and nitrogenous metabolism, considering a feature indicative of enhanced neuronal and metabolic activity. On the other hand, the upregulation of L-proline might suggest increased requirements for this amino acid, which is an important ingredient in protein synthesis and cellular signaling pathways besides the one involved in osmotic regulation. Furthermore, the apparent increase in choline phosphate reflects an increase in phospholipid synthesis important to the integrity and fluidity of cellular membranes. The distinct change pattern of CP_0.3 and CP_0.9 indicates dose-dependent effects on the metabolome profiles. There was a shift in the metabolite expression profiles in correspondence with an increased concentration of the treatment compound, further underlining that metabolic responses can occur frequently as a function of the therapeutic agent’s dosage. This has great importance for the optimization of therapeutic treatments since the results outline that the determination of proper dosing is needed to achieve the desired metabolic changes with minimum side effects (Figure 4A,B).

This is further complemented by the metabolic landscape given through variable importance in projection (VIP) analysis, pointing to important metabolites responsible for the major contribution to the difference between treatment groups. Particularly, metabolites with very high VIP scores were separated, including 3,4-dihydroxyphenylglycol sulfate and a variety of phospholipid species, as critical candidates for further investigation. These metabolites were very prominent in the analysis of the data and could, therefore, serve as biomarkers of treatment efficacy and might thus provide insight into the metabolic mechanisms of these therapeutic effects observed in the treatment groups. Being able to identify specific metabolites showing consistent and significant changes across multiple conditions further increases the reliability of using them as biomarkers in the future. The expression profile analysis further completes the trend observed in the VIP, with a constant trend toward differential expression in the same direction as the observations derived from both heatmap and VIP analyses. The convergence of such data underlines the fact that these key metabolites are relevant for the study of metabolic changes induced by treatments and hence are good biomarkers of physiological changes induced by therapeutic intervention (Figure 4C). In all, the findings represent the holistic view of metabolic reprogramming due to treatments of CP, underlining biomarkers that deserve further investigation. These insights identify the efficient biomarkers as therapeutic in clinical settings.

### 2.4. Correlation Highlights Co-Regulated Pathways, Dominance of Cellular Processes, and Enriched Apoptosis-Related Pathways

**Figure 5:**
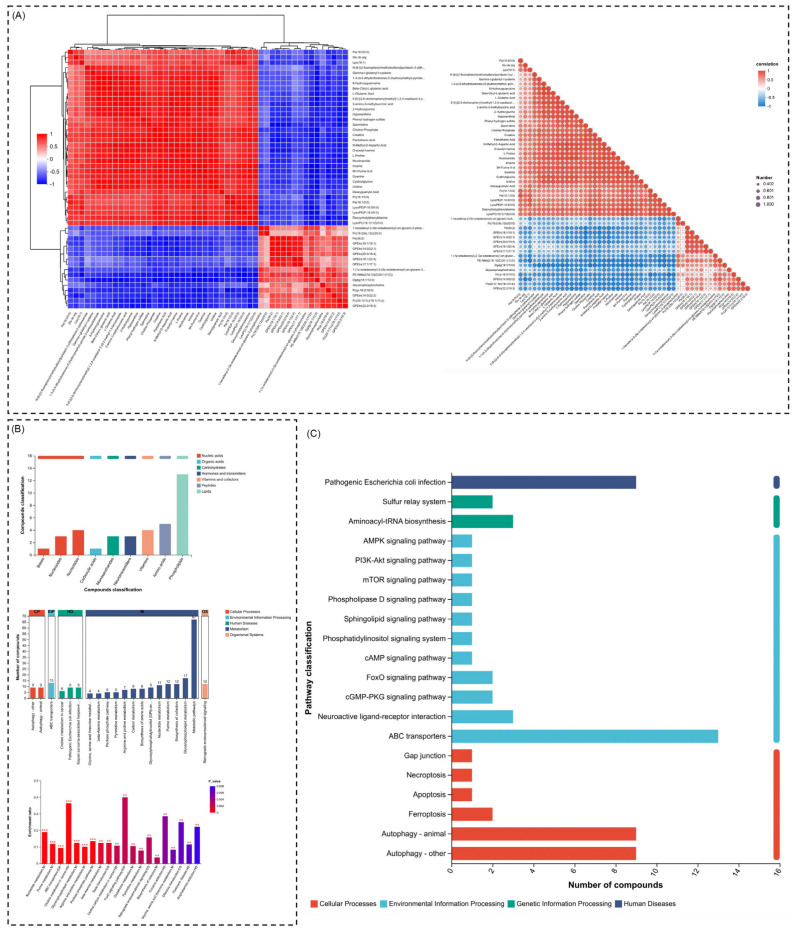
The overview of integrative analysis, highlighting compound pathway relationships, functional classifications, and pathway enrichments. (**A**) The heatmap displays the correlation structure between compounds and biological pathways, with hierarchical clustering segregating pathways into groups of positive (red) and negative (blue) correlations, emphasizing co-regulatory and inverse regulatory interactions. (**B**) Functional classification of identified compounds into categories such as cellular processes, genetic information processing, and human diseases, illustrating their predominant involvement in signaling and metabolic functions critical for cellular homeostasis. (**C**) Pathway enrichment analysis showcasing the distribution of compounds across key metabolic and signaling pathways, including apoptosis, AMPK signaling, and neuroactive ligand–receptor interactions, underlining their diverse biological significance and potential regulatory roles. “*” indicates statistical significance: *p* < 0.01 (**), and *p* < 0.001 (***).

A multifaceted approach combining correlation analysis, compound classification, and pathway enrichment allowed us to identify key metabolic and signaling pathways that are central to cellular functions and responses. The heatmap reveals both positive and negative associations among the pathways under investigation. One of the most notable findings is the robust positive correlation observed within the cellular signaling pathways, especially those involving AMPK signaling, mTOR signaling, and PI3K-Akt signaling. These pathways appear to be highly co-regulated, suggesting that they work in concert to control essential processes like cell growth, survival, and energy metabolism. Such co-regulation is consistent with the well-established roles of these pathways in maintaining cellular homeostasis, particularly in response to nutrient availability and stress conditions. The close alignment of these pathways indicates their synergistic roles in regulating cellular functions, with potential therapeutic implications for diseases like cancer and metabolic disorders (Figure 5A).

In contrast, we observed inverse correlations between stress response pathways such as autophagy and apoptosis with certain metabolic pathways. This suggests a finely tuned balance within the cell, where energy and metabolic processes may be downregulated under stress conditions to prioritize survival mechanisms such as cell death regulation and autophagic degradation of damaged cellular components. This finding supports the idea that cells dynamically adjust their metabolic networks in response to internal and external challenges, balancing growth and survival functions against cellular integrity maintenance. The clustering of these pathways further highlights the need for understanding how the cellular response to stress can be either protective or deleterious, depending on the context. Moving on to compound class enrichment, cellular processes were the most dominant class, integrating pathways of autophagy, apoptosis, and protein folding. Especially, compounds associated with autophagy were the most enriched, mainly those involved in lysosome degradation and mitophagy. This is consistent with recent studies that point out the critical role of autophagy for cellular homeostasis, in particular under stress conditions in which cells need to degrade dysfunctional organelles or proteins. Accordingly, the high enrichment of compounds in the autophagic pathways indicated that these compounds likely function as modulators of the autophagic flux and thus may be potential drugs against diseases caused by defective autophagy, including neurodegenerative diseases and cancer (Figure 5B). Furthermore, the high ranking of compounds associated with apoptosis highlights the importance of regulated cell death in homeostasis. Also, targeting compounds associated with active pathways of caspase activation and mitochondrial permeability transition indicates that these pathways are active in cellular homeostasis, especially when oxidative stress or DNA damage needs to be overcome. These data further support the concept that apoptosis is tightly regulated to avoid tumorigenesis as well as excess cell death in degenerative diseases.

In line with this, the enrichment study highlights pathways related to cellular survival and metabolic processes, including AMPK signaling, mTOR signaling, and the PI3K-Akt signaling pathway, as the most enriched. These pathways bear vital relevance to energy homeostasis, though they interface at large with neuroactive ligand–receptor interactions and sulfur relay systems, reflecting an interwoven complexity of the cellular communication network. Identification of such pathways as crucial compound-enriched nodes now suggests that the compounds studied here will very likely affect many facets of cellular function, such as cell–cell communication, nutrient sensing, and stress adaptation. More significantly, enrichment of the neuroactive ligand–receptor interaction pathways further points towards the fact that such compounds may modify neurological functions as well. As a large number of compounds were found to be associated with these pathways, it is nothing less than probable that these molecular entities hold a significant position in the realm of cellular communications and neuronal signaling and, hence, may affect neurotransmitter systems and synaptic plasticity. The presence of relay systems with sulfur further supports the notion that cellular redox states and sulfur metabolism are of great importance in the health and function of the cell, especially in the response to oxidative stress (Figure 5C). All these pathways interlace into a network of co-regulated processes underlining their importance for the maintenance of cellular integrity in conditions of change. Taken together, these data allow one to envision compounds possibly playing a major role in regulating cellular metabolism, survival, and response to stress. Thus, understanding exactly how such pathways interacting at the molecular level may therefore lead to targeted therapies able to modulate critical functions in living cells.

### 2.5. Metabolic Reprogramming and Oxidative Stress Regulation Highlighted Through Pathway Enrichment and Compound Analysis

The findings reveal a profound interplay between metabolic reprogramming and oxidative stress regulation, providing key insights into the dynamic responses of cellular systems under specific conditions. By analyzing pathway enrichment, differential abundance, and compound classifications, we uncover critical shifts in metabolic pathways and stress-related processes that underscore the cellular adaptations to external or internal perturbations. A multi-dimensional analysis of compound pathway enrichments, differential abundance scores, and compound classifications, shedding light on the biological processes and metabolic networks most influenced by the identified compounds, was carried out. The findings highlight specific pathways that are functionally enriched and dominant under different experimental conditions, offering significant insights into cellular regulation and metabolic adaptability.

**Figure 6:**
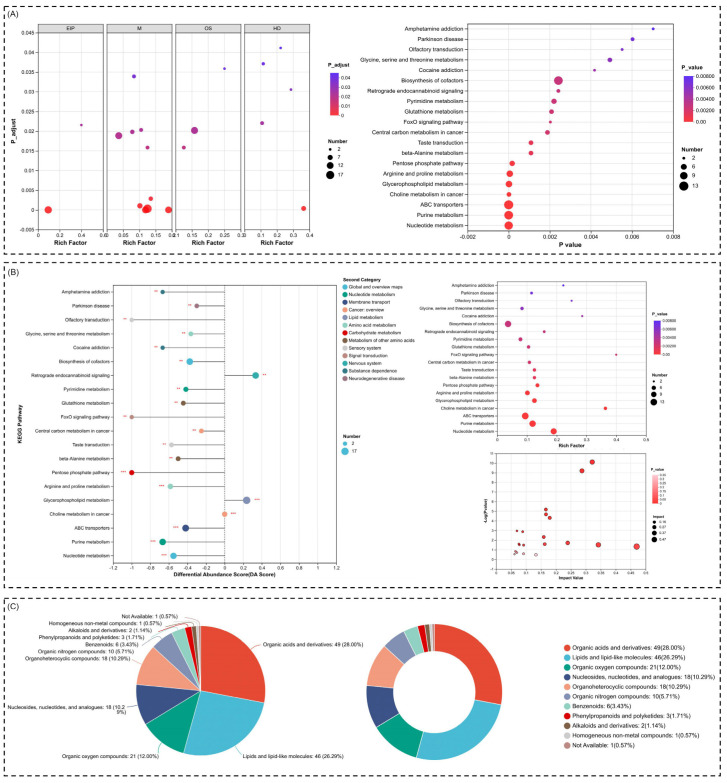
A detailed multi-level analysis of pathway enrichment, differential abundance, and compound classifications. (**A**) Pathway enrichment across categories, with significant pathways including glycine, serine, threonine metabolisms, glutathione metabolism, and Parkinson’s disease pathways. (**B**) Differential abundance analysis underlining pathways with more changes, pointing to central carbon metabolism, amino acid turnover, and oxidative stress pathways. (**C**) A classification of the compounds focusing on organic acids and derivatives, lipids, and nucleotides, revealing the central role in metabolism, redox regulation, and cellular signaling. Disease-associated pathways (for example, Parkinson’s disease) are KEGG-based classifications indicating biochemical homologies, not causal consequences, in the MDBK cell model. “*” indicates statistical significance: *p* < 0.01 (**), and *p* < 0.001 (***).

We found a very clear distinction in pathway enrichment across categories, with metabolism pathways being the most significant, particularly glycine, serine, and threonine metabolisms, pyrimidine metabolism, and glutathione metabolism. These results are indicative of the critical roles that these pathways play in the production of energy, synthesis of nucleotides, and redox balance, respectively. The fact that glycine, serine, and threonine metabolisms are among the most enriched signifies their importance in amino acid synthesis and one-carbon metabolism, which is very fundamental in the biosynthesis of both nucleotides and proteins. Pyrimidine metabolism supports this relationship, considering that nucleotides are in higher demand under certain conditions, possibly as a mechanism to sustain DNA and RNA synthesis during cell stress or growth phases. Glutathione metabolism reflects the cellular response to oxidative stress, as it is a major antioxidant protecting against oxidative damage by helping maintain redox homeostasis. KEGG-defined human disease pathways, for instance Parkinson’s disease and amphetamine dependence, are also highly enriched. These annotations capture non-causal, homology-derived relationships and mark convergence in biochemical components or stress-related molecular properties. Such enrichment indicates shared metabolic motifs but does not imply occurrence or emergence of human disease in bovine cells. The enrichment of Parkinson’s disease pathways underscores the role of oxidative stress, mitochondrial dysfunction, and impaired energy regulation, which are hallmarks of neurodegenerative processes. Additionally, pathways related to central carbon metabolism, arginine and proline metabolism, and beta-alanine metabolism suggest alterations in energy production and amino acid turnover, potentially to meet the demands of stressed or dysregulated cells. Notably, Parkinson’s disease, glutathione metabolism, and retrograde endocannabinoid signaling pathways demonstrate high enrichment and low *p*-values. This reinforces the importance of oxidative stress regulation and metabolic signaling in cellular function. Retrograde endocannabinoid signaling may indicate a role for lipid mediators in modulating cellular processes such as inflammation and neurotransmission, particularly under disease conditions. The biosynthesis of cofactors and nucleotide metabolism pathways further emphasizes the active role of these compounds in supporting essential cellular functions like enzyme activity and genetic information maintenance (Figure 6A). On exploring differential abundance scores for KEGG pathways, we identified pathways with the greatest shifts in abundance. Notably, glycine, serine, and threonine metabolisms again emerge with significant positive scores, indicating their elevated activity compared to other pathways. Other pathways, such as glutathione metabolism, retrograde endocannabinoid signaling, and pentose phosphate pathways, are similarly overrepresented, suggesting a dynamic interplay between redox regulation, amino acid metabolism, and energy production. Interestingly, the enrichment of central carbon metabolism highlights the broader activation of core metabolic pathways necessary for cellular survival and proliferation. However, there is a consistent enrichment of metabolism-related pathways, with arginine and proline metabolism, glutathione metabolism, and central carbon metabolism, appearing as prominent nodes. This reinforces the importance of metabolic reprogramming under the studied conditions, potentially to optimize cellular energy and biomolecule synthesis while counteracting oxidative stress. The link between amino acid metabolism and energy pathways further suggests a coordinated response to nutrient availability and cellular stress (Figure 6B).

Later, we organized compounds into functional classes, providing insight into their chemical diversity and biological relevance. Organic acids and derivatives account for the largest proportion, comprising approximately 28%, followed closely by lipids and lipid-like molecules. This suggests that many of the identified compounds play central roles in intermediary metabolism and cellular signaling, particularly through lipid-mediated pathways. The significant presence of organic oxygen compounds and nucleosides, nucleotides, and analogs further highlights their roles in redox balance, nucleotide synthesis, and cellular respiration. Compounds related to organoheterocyclic structures and amino acid derivatives underscore the active involvement of nitrogen-based molecules in metabolic and signaling processes (Figure 6C).

Taken together, the findings reveal a comprehensive network of enriched pathways and chemical compounds that are closely intertwined. The consistent enrichment of glycine, serine, and threonine metabolisms, glutathione metabolism, and nucleotide synthesis pathways highlights the critical balance between biosynthesis, redox regulation, and energy production under specific biological conditions. The enrichment of disease-related pathways like Parkinson’s disease also connects these processes to neurodegenerative mechanisms, suggesting that oxidative stress and metabolic reprogramming may underlie disease pathology. These insights collectively point to the interconnected roles of metabolism, amino acid turnover, and stress response in maintaining cellular function and addressing external or internal challenges.

## 3. Discussion

The pharmacokinetic interaction between the antimalarial drugs and their metabolites supports dose-dependent metabolic shifts. Previous studies have put into perspective how dihydroartemisinin (DHA) acts as the active metabolite of artemisinin-based compounds, driving such metabolic effects [12,13,14]. Conversion of artemisinin derivatives, such as artesunate and artemether, to DHA might influence the overall metabolic response in a dose-dependent manner. In our current findings, unique metabolites were detected in the treated groups, CP and CP_D, when compared to the controls. Such a complex interaction of antimalarial drugs with various metabolic pathways could be one of the reasons. Previous studies have shown that antimalarial drugs, including dihydroartemisinin, may modulate the activity of the key enzymes responsible for drug metabolism, including cytochrome P450 (CYP) isoforms, resulting in altered metabolite profiles [15]. The robustness of the PLS-DA model validated by permutation testing was similarly carried out in other metabolomic studies of antimalarial drugs. This statistical rigor is important in making the metabolic signatures identified reliable and establishing their potential as biomarkers for the efficacy of treatments [16].

Apart from that, the dose-dependent metabolic changes in this study might have broader implications in terms of detailing mechanisms of action and possible therapeutic uses for dihydroartemisinin. The latter, according to previous reports, was demonstrated to be able to modulate various cellular processes related to energy metabolism, cell growth, and apoptosis; these differences could ultimately reflect in changed metabolomic profiles [17,18]. In addition, pharmacokinetic interactions among antimalarial drugs with other co-medications in this study, like antiretroviral therapies, would determine some changes seen in the metabolome. Drug–drug interactions, in fact, may indeed impact the disposition and metabolism of antimalarial compounds themselves, hence probably resulting in differential metabolic signatures [19,20]. Lastly, the metabolomics approach undertaken in this study follows a growing body of evidence underlining the value of metabolomics to decipher complicated host–parasite relationships and, particularly, to provide insight into the mechanisms of action of antimalarial drugs; the identification of unique and shared metabolites could thus generate unique information regarding the basic mechanisms responsible for antimalarial drug response and resistance [21].

Moreover, we observed a strong upregulation of phospholipids and fatty acids, indicative of altered lipid metabolism. Indeed, lipid metabolism has been found to be critical for the maintenance of cellular integrity and energy homeostasis, especially during stressful conditions. For instance, a researcher showed that disruption in lipid metabolism in bovine adipose tissues results in increased vulnerability to metabolic disorders, showing how lipid homoeostasis is key to animal health optimality [22]. The upregulation of phospholipids in our study suggests that there could be membrane remodeling. Other studies have shown the metabolic responses of bovine liver under heat stress [23,24]. This underlines adaptive mechanisms that bovine cells use to meet the challenge of stress through altering membrane composition in order to maintain fluidity and functionality. The differential expression of amino acids, including L-lysine and L-ornithine, indicates considerable changes in protein metabolism. Amino acids are critical in multiple biological processes, from protein synthesis and signaling to energy production. Thus, L-lysine enrichment was found to be consistent with prior studies indicating that dietary supplementation improves metabolic health and growth potential in cattle [25,26]. This indicates that the critical availability and metabolism of amino acids are important factors to further promote livestock productivity. Additionally, vital changes in nitrogen metabolism further depress overall animal health and productivity [27].

These metabolomic profiles are strongly correlated with the known mechanistic impacts of dihydroartemisinin, most prominently its capacity to influence oxidative stress and mitochondrial dysfunction [28], which is supported by our enrichment of glutathione metabolism and the substantial variation in redox-related amino acids such as L-glutamic acid and L-proline. The current research also noted substantial enrichment of stress signaling pathways such as AMPK, PI3K-Akt, and mTOR signaling cascades, which are generally activated in response to mitochondrial stress and cellular energy imbalance [29]. Additionally, elevated levels of phospholipid species and choline phosphate reveal membrane remodeling, in agreement with ROS-mediated lipid turnover and mitochondrial adaptation. Therefore, our results establish a mechanistic connection between exposure to DHA and the metabolic stress response it induces in MDBK cells.

Further enrichment analysis of the pathways explained how these changes in metabolism affect cellular function. The identified enrichment of arachidonic acid metabolism is critical in producing bioactive lipids and regulating inflammatory responses and cell signaling. Alterations in arachidonic acid metabolism have been shown in previous studies to be associated with the pathophysiology of a wide range of diseases in livestock; mastitis and metabolic syndrome were among the diseases related to changes in this metabolite flux [30,31]. An increase in the production of bioactive lipids can be part of an adaptive response to stress, which likely promotes recovery from disturbances of metabolism. The knowledge of these pathways can be important for the development of nutritional strategies that enhance livestock resilience to stressors. Another major identification was the significant involvement of the metabolism of nucleotides, primarily purine synthesis. Alteration in cellular energy homeostasis may result in metabolic imbalance and poor cellular functions. This is in agreement with other studies showing that changes in bovine liver cell nucleotide metabolism were linked with increased levels of oxidative stress and also with metabolic impairment [32,33]. Metabolic health consequences of these findings go beyond the fact that dietary manipulations targeting metabolic health improve the ability of livestock to handle environmental and physiological stressors. Related to the changes in lipid and amino acid metabolism, other findings on carboxylic acids further point to changes in metabolism that occur in response to treatment. The dominance of carboxylic acids and their derivatives supports a key role in energy production and biosynthesis. Many studies have stressed the importance of these metabolites in maintaining energy homeostasis and in enabling metabolic reprogramming under different conditions [34]. Since the metabolic change in carboxylic acids corresponds with the energy needs of bovine kidney cells under stress, it calls for nutritional interventions targeted to meet optimal energy metabolism in livestock. This underlines the fact that the treatment concentration is relevant for dose-dependent effects on metabolite expression patterns. The multivariate analyses have clearly differentiated the CP and CP_D groups from each other and thus have lent support to earlier reports on considerable differences in metabolic response based on dosage [35]. In therapeutic applications, dosing regimens must be given due care so that interventions induce desired beneficial metabolic changes with minimum side effects. In addition, the present study points out specific metabolites as markers of the therapeutic effect. This would also open up further research towards tailoring treatment protocols that will bring about maximal improvement in metabolic health. Furthermore, in this study, correlation analysis unraveled co-regulated pathways controlling cellular functions. The key signaling pathways like AMPK, mTOR, and PI3K-Akt show positive correlations, thus pointing toward an integrated response to metabolic perturbations. These are documented literature-wise as central regulators of cellular metabolism, growth, and survival processes [36,37,38]. This co-regulation of pathways reflects the interconnectivity of metabolic networks and puts emphasis on the holistic understanding needed in cellular responses in livestock management. These findings have also shown how tightly related apoptosis, autophagy, and metabolic pathways are with each other. The negative correlations suggest that when stress exists, cells may emphasize survival machineries such as autophagy rather than producing energy. This is in line with evidence, which has shown that autophagy protects cellular integrity during metabolic stress [39]. The understanding of the interaction among these pathways could help define strategies for the improvement of stress tolerance in livestock to improve welfare and productivity. The current study employs a comprehensive untargeted metabolomics platform with validated statistical modeling (PLS-DA and permutation testing) to support data reproducibility and reliability. Dose-responsive analyses provide increased granularity into the bioactivity of DHA, and pathway enrichment and VIP analyses provide increased mechanistic insight into metabolic modulation by DHA. As for limitations of the study, there are concerns regarding that in vitro observations were conducted with a single cell line, which may not capture the entire complexity of systemic responses in vivo. Moreover, although metabolomics yields strong correlational data, additional functional validation (e.g., gene/protein expression assays) would enhance mechanistic interpretation.

## 4. Materials and Methods

### 4.1. Sample Source and Preparation

Madin-Darby bovine kidney (MDBK) cells (CCBC071) used in the study were provided by the China Food and Drug Administration. The cell line was grown in a sterile environment in DMEM with 10% FBS and 1% penicillin–streptomycin. The cells were developed in a humid incubator at 37 °C and 5% CO_2_. For experimental treatments, cells were seeded in 6-well plates and allowed to attach and proliferate to 70–80% confluence. Cells were segregated into three experimental groups: a control (CON), a low-dose treatment (CP_0.3), and a high-dose treatment (CP_0.9) group. At desired confluence, the control group received no additional compounds in serum-free media, while experimental groups were treated with CP, respectively, at 0.3 µM and 0.9 µM for 24 h. The particular dosing concentrations were selected on prior optimization studies that intended to identify dose-dependent metabolic dynamics. To remove residual serum-derived metabolites, cells were washed twice with phosphate-buffered saline (PBS) prior to treatment. After incubation, cells were trypsinized, centrifuged at 1000× *g* for 5 min, and snap-frozen in liquid nitrogen. Cell pellets were stored at −80 °C to preserve the metabolome during harvesting.

### 4.2. Metabolite Extraction

An 80:20 *v*/*v* methanol/water solution was prepared in order to optimize the solubilization of both polar and non-polar metabolites for the extraction of metabolites. For every cell pellet, containing about 1 × 10^6^ cells, 200 µL of the extraction solvent was added. The mixture was then homogenized with a bead mill for 3 min to fully break down the cellular matrix, followed by 10 min of sonication in an ultrasonic bath, which was kept ice-cold to ensure maximum release of metabolites. The samples were centrifuged at 13,000× *g* for 15 min at 4 °C; then, the supernatant was vacuum-dried. The dried metabolites were reconstituted in 50% methanol for further LC-MS analysis. The QC samples have passed through the same processes given to experimental and control samples for consistency in the analysis.

### 4.3. LC-MS Analysis

Metabolomics profiling was executed by LC-MS on an HPLC coupled to a QTOF mass spectrometer. Chromatographic separation was executed on a C18 reverse-phase column (dimensions: 100 × 2.1 mm, 1.8 µm particle size) at a controlled temperature of 40 °C to improve the peak shape and resolution. The mobile phase used for chromatographic separation consisted of two solvents: solvent A, 0.1% formic acid in water, and solvent B, 0.1% formic acid in acetonitrile. In an effort to permit the elution of hydrophilic metabolites, gradient elution was performed by starting the elution with 95% A and 5% B in the first 5 min. The gradient then progressed to 10% A and 90% B in 25 min. Afterwards, column conditions were returned to their initial state (95% A, 5% B), and re-equilibration was allowed for an additional 5 min prior to the next injection. For comprehensive detection of a wide range of metabolites, mass spectrometric detection was performed in both positive and negative ionization modes. Targeted MS/MS authentication was carried out for a few key metabolites with authentic standards, such as L-glutamic acid, choline phosphate, and lysophosphatidylcholine (18:0), to reach Level 1 identification confidence. Standards were run under similar chromatographic and spectrometric circumstances to allow retention time and fragmentation spectrum comparison. The source temperature was controlled at 350 °C with a capillary voltage of 3.5 kV to optimize ionization. Data were collected within the mass range of *m*/*z* 100–1500. At the start and finish of every analytical batch, quality control standards were analyzed to verify instrument stability and reproducibility during the course of the analysis.

### 4.4. Data Preprocessing

Raw LC-MS data were analyzed by proprietary software developed by Majorbio to handle peak detection, alignment, and normalization processes specific for metabolomics data processing. The raw data preprocessing included the following steps: Missing values in the dataset were treated using median imputation so that the crucial information would not be lost. RSDs above 30% in QC samples were therefore excluded from further analysis on the basis that high values of RSD imply a variance in the measurements that could either be due to instrument instability or sample degradation. The remaining data underwent log transformation to correct for non-normality and reduce the effect of outliers for preparation prior to statistical analysis in order to keep the data normally distributed.

### 4.5. Statistical Analysis

Univariate statistical analysis was used to identify those metabolites that were significantly different between the experimental and control groups. To that end, for each metabolite, a Student’s *t*-test was carried out, with the threshold of the *p*-value set below 0.05. Moreover, FC criteria were utilized to screen for metabolites whose expression showed striking variations, considering an FC value greater than 2 or less than 0.5. Multivariate statistical methods were applied for a more comprehensive analysis, including principal component analysis (PCA) and partial least squares discriminant analysis (PLS-DA). PCA was conducted to assess the overall clustering and visualize the variance. PLS-DA was utilized to identify the metabolites that contributed significantly to the separation between the experimental and control groups. Model validation of PLS-DA was performed by permutation testing to ensure overfitting was not causing the observed separation between the groups.

### 4.6. Pathway Analysis

The differentially expressed metabolites were mapped to KEGG and HMDB databases for pathway enrichment analysis, in order to identify which metabolic pathways had been significantly affected by the experimental treatments. In order to bolster pathway analysis confidence, key metabolites like glutamic acid and choline phosphate were verified by MS/MS spectral matching and retention time alignment to reference standards. Pathway enrichment analysis was performed based on Fisher’s exact test, and the pathways with a *p*-value less than 0.05 were noted as significant. The metabolic pathways have been visualized using software tools, enriched by the identified metabolites. This enabled the detection of the main biological processes and pathways modulated by the experimental condition.

### 4.7. Visualization and Bioinformatics

The interpretation of the data has been employed to develop a variety of visualization techniques, including heatmaps, volcano plots, and Venn diagrams, using Python and R packages (V3.10). Heatmaps were used to show the metabolite expression pattern in different groups. In addition, volcano plots and Venn diagrams were used to show the statistical significance versus fold change of unique and shared metabolites to identify significantly changed metabolites. Networks of correlation have been built with a view to establishing relationships among metabolites to eventually explain the biological meaning of the metabolic variations observed. Significant pathway enrichment was visualized using KEGG Mapper and iPath 3.0 in order to highlight the metabolic processes that were significantly influenced by applying experimental treatments.

## 5. Conclusions

The metabolomic profile of bovine kidney cells reveals how dihydroartemisinin (DHA) affects cellular metabolism in the field of phytopharmacology, affirming its status as a metabolic modulator. The observed changes in lipid reorganization, amino acid metabolism, and nucleotide biosynthesis reflect the vast array of biochemical changes caused by DHA, emphasizing its significance in phytochemical-controlled metabolic processes. Overall, these findings advance the elucidation of herbal bioactive compounds in cellular homeostatic modulation and stress regulation mechanisms, thus affirming their potential in disease model-targeted modulation of metabolic well-being. Follow-up research must focus on elucidating the pharmacokinetic characteristics of DHA and related pharmacotherapeutic utility in disease model scenarios in view of its potential to protect against metabolic dysfunctions as well as counter environmental stresses by modulation through phytochemistry.

## Data Availability

The data supporting the findings of this study, titled Metabolomic Profiling of Madin-Darby Bovine Kidney Cells, are available in the BioProject repository under the accession number PRJNA1212795 https://www.ncbi.nlm.nih.gov/bioproject/PRJNA1212795 (accessed on 20 January 2025). The processed data associated with this project can be accessed through the NCBI Sequence Read Archive (SRA) with the submission ID SUB14325819.
